# GriddingMachine, a database and software for Earth system modeling at global and regional scales

**DOI:** 10.1038/s41597-022-01346-x

**Published:** 2022-06-01

**Authors:** Yujie Wang, Philipp Köhler, Renato K. Braghiere, Marcos Longo, Russell Doughty, A. Anthony Bloom, Christian Frankenberg

**Affiliations:** 1 Division of Geological and Planetary Sciences, California Institute of Technology, 1200 E California Blvd, Pasadena, 91125 California USA; 2 Jet Propulsion Laboratory, California Institute of Technology, 4800 Oak Grove Dr, Pasadena, 91109 California USA; 3 Joint Institute for Regional Earth System Science and Engineering, University of California, Los Angeles, 405 Hilgard Avenue, Los Angeles, 90095 California USA; 4 Climate and Ecosystem Sciences Division, Lawrence Berkeley National Laboratory, 1 Cyclotron Rd, Berkeley, 94720 California USA; 5 College of Atmospheric & Geographic Sciences, University of Oklahoma, 660 Parrington Oval, Norman, 73019 Oklahoma USA

**Keywords:** Carbon cycle, Biogeochemistry

## Abstract

Land and Earth system modeling is moving towards more explicit biophysical representations, requiring increasing variety of datasets for initialization and benchmarking. However, researchers often have difficulties in identifying and integrating non-standardized datasets from various sources. We aim towards a standardized database and one-stop distribution method of global datasets. Here, we present the GriddingMachine as (1) a database of global-scale datasets commonly used to parameterize or benchmark the models, from plant traits to vegetation indices and geophysical information and (2) a cross-platform open source software to download and request a subset of datasets with only a few lines of code. The GriddingMachine datasets can be accessed either manually through traditional HTTP, or automatically using modern programming languages including Julia, Matlab, Octave, Python, and R. The GriddingMachine collections can be used for any land and Earth modeling framework and ecological research at the regional and global scales, and the number of datasets will continue to grow to meet the increasing needs of research communities.

## Introduction

Land components in Earth system models (ESMs) are moving towards more explicit biophysical representation. For example, in the modeling of the soil-plant-air continuum, vegetation canopy complexity has increased from a single big-leaf to a multi-layered modeling approach including leaf angular distributions^1^, thus allowing for more realistic and predictive simulations, bridging Earth surface processes with remote sensing^2^. Further, the use of plant trait- and process-based stomatal models are also drawing more attention given the improved predictive skills compared to empirical representations^3–5^ and more direct link to plant physiological status^6^. However, implementing these biophysical representations globally has been challenging due to the lack of a complete suite of global soil and plant traits and the difficulties to assess model simulations.

The capability of recently developed terrestrial biosphere models in simulating plant physiological processes and canopy optical properties and bridging them to remote sensing makes it possible to constrain Earth surface processes using remote sensing data^7–10^. Notably, increasing volume and types of global scale ecological datasets, products, and databases are largely being made publicly available in recent years, such as multiple decades of satellite-based maps of vegetation indices from Moderate Resolution Imaging Spectroradiometer (MODIS) instruments. These global scale datasets may serve as initial or boundary conditions or as benchmark standards for the ESMs^11,12^.

However, in parallel to the great promise of the ever-increasing volume of data, harnessing these datasets is becoming a considerable challenge for the research communities. Typical day-to-day challenges for researchers emerge because the datasets (1) are posted and stored at different websites; (2) have different formats (e.g., GeoTIFF and NetCDF files); (3) may not be usable directly out of the box (e.g., data is converted to Bytes for smaller size, and thus re-scaling is required); (4) have different orientations (e.g., some from Southern to Northern Hemisphere, and vice versa); (5) have different latitude and longitude setups (e.g., some exclude high latitude regions); (6) have different projections (e.g., cylindrical and pseudo-cylindrical); and (7) may have different and non-standard units. These limitations pose substantial barriers even for experienced programmers, given the time required to find and reformat the data. Particularly, it is very inconvenient for both beginners and experienced researchers who may just require a tailored subset of these broadly available scientific datasets. For instance, one may need to download the entire global dataset to obtain information for a few sites or a small region.

The emergence of the Google Earth Engine (GEE)^13^ has largely advanced data sharing and reuse, and the cloud computing platform provided further relaxes the need for extensive local storage and computation resource. However, GEE is not always the best solution for researchers given that users do not have direct control over the datasets for easier offline analysis and debugging. Most importantly, many public datasets circulate within the research communities but are not converted to GEE images. In comparison, the Application for Extracting and Exploring Analysis Ready Samples (AppEEARS) and European Centre for Medium-Range Weather Forecasts (ECMWF) offer users simple and efficient ways to request raw data subsets (limited to their own datasets; need to register, and download and manage the subsets manually). Therefore, it remains a question how to more effectively share, manage, and reuse these community-based datasets stored on local hard drives. One way to circumvent this issue is to build a database for these community datasets and label each with a unique easy-to-read tag. Using tags to automatically download and manage the datasets would largely simplify the user end operations and facilitate the data reuse.

**Fig. 1:**
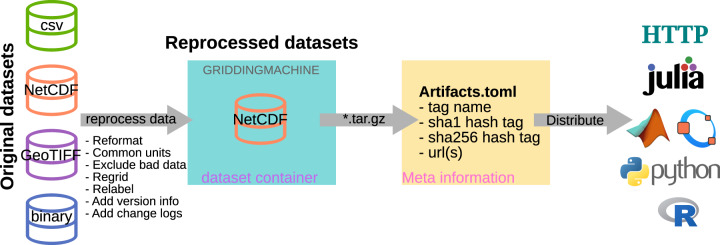
Pathway used to assemble and distribute the GriddingMachine database. Each dataset is (**a**) reprocessed to meet our standards for distribution, (**b**) compressed as a tar.gz file along with an empty label file GRIDDINGMACHINE, and (**c**) stored on publicly available HTTP servers. Then the meta information for each dataset is stored in Artifacts.toml, which includes the tag name, sha1 hashtag, sha256 hashtag, and downloading URLs. Users are able to access the datasets manually via HTTP protocols or automatically through Julia, Matlab, Octave, Python, and R functions aided by Artifacts.toml.

Our aim is to create and maintain such a standardized collection of globally spanning datasets for biophysical representations in ESMs. Therefore, we need to resolve the problems listed above and distribute standardized datasets using tags. Our solution, GriddingMachine, is a one-stop shop for downloading, storing, exploring, and jointly extracting multiple datasets we have collected and reprocessed. GriddingMachine was initially developed in the Julia programming language^14^ to parameterize a new-generation ESM developed by the Climate Modeling Alliance (CliMA), particularly the land component—CliMA Land^9,15,16^. We have further generalized the model for the purpose of processing and distributing datasets by adding more global scale datasets other than the land parameters (such as remote sensing based products), and providing more user interfaces through HTTP, Matlab, Octave, Python, and R in addition to Julia (Fig. 1).

## Results

Each dataset in GriddingMachine database is labeled with a unique tag following a general naming pattern: LABEL_(EXTRALABEL_)IX_JT_(YEAR_)VK, where EXTRALABEL and YEAR are optional (Table 1). Here, LABEL is the major data identifier (e.g., GPP for gross primary productivity), EXTRALABEL is the secondary data identifier to distinguish datasets within a collection (for example, GPP from different models), IX indicates the spatial resolution is 1/I°, JT represents the temporal resolution (H for hour, D for day, M for month, and Y for year), and VK means the dataset version within our collection (V1 for data from publication 1, and VN for data from publication N). For example, LAI_MODIS_20X_1 M_2008_V1 stands for leaf area index (LAI) from MODIS at 1/20° spatial resolution and 1 month temporal resolution for the year 2008, and V1 indicates that the dataset is the first LAI collection; VCMAX_2X_1Y_V2 stands for maximum carboxylation rate (*V*_*cmax*_) at 1/2° spatial resolution and 1-year temporal resolution, and V2 indicates that the dataset is the second *V*_*cmax*_ collection.

**Table 1:** Datasets within GriddingMachine collections.

Dataset type	LABEL	EXTRALABEL	IX	JT	YEAR	VK	Reference
Biomass	BIOMASS	ROOT	120X	1Y	—	V1	^20^
	BIOMASS	SHOOT	120X	1Y	—	V2	^21^
Canopy height	CH	—	20X	1Y	—	V1	^22^
	CH	—	2X	1Y	—	V2	^23^
Clumping index	CI	—	2X, 240X	1Y	—	V1	^24^
	CI	—	2X	1Y	—	V2	^25^
Elevation	ELEV	—	4X	1Y	—	V1	^26^
Gross primary productivity	GPP	MPI_RS	2X	1 M, 8D	2001–2019	V1	^27^
	GPP	VPM	5X, 12X	8D	2000–2019	V2	^28^
Leaf area index	LAI	MODIS	2X, 10X, 20X	1 M, 8D	2000–2020	V1	^29^
Land mask	LM	—	4X	1Y	—	V1	ERA5
Leaf nitrogen content	LNC	—	2X	1Y	—	V1	^30^
	LNC	—	2X	1Y	—	V2	^23^
Leaf phosphorus content	LPC	—	2X	1Y	—	V1	^30^
Plant functional type	PFT	—	2X	1Y	—	V1	^31^
Surface area	SA	—	1X, 2X	1Y	—	V1	^31^
Soil color class	SC	—	2X	1Y	—	V1	^31^
Solar-induced chlorophyll fluorescence	SIF	TROPOMI_740	1X, 2X, 4X, 5X, 12X	1 M, 8D	2018–2020	V1	^18^
	SIF	TROPOMI_740DC	1X, 2X, 4X, 5X, 12X	1 M, 8D	2018–2020	V1	^18^
	SIF	TROPOMI_683	1X, 2X, 4X, 5X, 12X	1 M, 8D	2018–2020	V2	^19^
	SIF	TROPOMI_683DC	1X, 2X, 4X, 5X, 12X	1 M, 8D	2018–2020	V2	^19^
	SIF	OCO2_757	5X	1 M	2014–2020	V3	^32^
	SIF	OCO2_757DC	5X	1 M	2014–2020	V3	^32^
	SIF	OCO2_771	5X	1 M	2014–2020	V3	^32^
	SIF	OCO2_771DC	5X	1M	2014–2020	V3	^32^
Solar-induced luminescence	SIL	—	20X	1Y	—	V1	^33^
Specific leaf area	SLA	—	2X	1Y	—	V1	^30^
	SLA	—	2X	1Y	—	V2	^23^
Soil hydraulics	SOIL	SWCR	12X, 120X	1Y	—	V1	^34^
	SOIL	SWCS	12X, 120X	1Y	—	V1	^34^
	SOIL	VGA	12X, 120X	1Y	—	V1	^34^
	SOIL	VGN	12X, 120X	1Y	—	V1	^34^
	SOIL	KSAT	100X	1Y	—	V2	^35^
Tree density	TD	—	2X, 120X	1Y	—	V1	^36^
Maximum carboxylation rate	VCMAX	—	2X	1Y	—	V1	^37^
	VCMAX	—	2X	1Y	—	V2	^38^
Wood density	WD	—	2X	1Y	—	V1	^23^

Although we aim for automatic and convenient data access within diverse programming languages, we still provide traditional data access from HTTP servers. The compressed datasets can be manually downloaded from an open data archive hosted on CaltechDATA^17^.

### Julia support

**Fig. 2:**
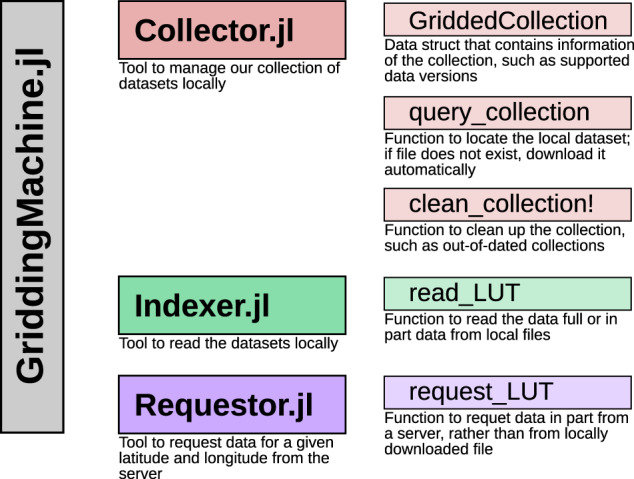
Framework of GriddingMachine.jl package (v0.2). GriddingMachine.jl contains three sub-modules: Collector, Indexer, and Requestor. Collector downloads and manages the datasets; Indexer reads the downloaded datasets; and Requestor requests a subset of data directly from the server without downloading the datasets.

We provide Julia language support using GriddingMachine.jl (v0.2, requires Julia 1.6+), which include three tested sub-modules: Collector, Indexer, and Requestor (Fig. 2). Collector is responsible for downloading and managing datasets; Indexer is responsible for reading downloaded datasets; and Requestor is responsible for requesting a subset of data directly from our server without downloading the datasets. To install GriddingMachine.jl, one may simply type the following in Julia REPL (read-eval-print loop):

Below, we introduce only the fundamental features of GriddingMachine.jl, and refer the readers to our online documentation for more details about GriddingMachine.jl at https://clima.github.io/GriddingMachine.jl/stable/.

### Download data

We classify the datasets into different collections using the GriddedCollection structure, which includes the following fields:

- LABEL: a string that appears in file names, such as LAI;
- SUPPORTED_COMBOS: an array of string for supported combinations, such as [MODIS_2X_8D_2018_V1, MODIS_2X_8D_2019_V1, MODIS_2X_8D_2020_V1];
- DEFAULT_COMBO: a string of the default combination, such as MODIS_2X_8D_2020_V1.

Currently, we have a total of 19 categories of dataset (column Dataset type in Table 1), and 19 functions to construct these GriddedCollection structures accordingly:

- biomass_collection
- canopy_height_collection
- clumping_index_collection
- elevation_collection
- gpp_collection
- lai_collection
- land_mask_collection
- leaf_nitrogen_collection
- leaf_phosphorus_collection
- pft_collection
- sif_collection
- sil_collection
- sla_collection
- soil_color_collection
- soil_hydraulics_collection
- surface_area_collection
- tree_density_collection
- vcmax_collection
- wood_density_collection

For example, one can define a LAI collection and check supported combinations using the following commands:

Function query_collection returns the path to the dataset (Julia will download the file automatically to folder ~/.julia/artifacts if the dataset does not exist):

The first method returns the path to default combination; and the second and third methods return the path to target dataset.

Function clean_collection! cleans up the downloaded datasets:

The first and second methods clean up all outdated datasets that were not in Artifacts.toml; the third method cleans up all GriddingMachine datasets; the fourth method cleans up all selected datasets; and the last method cleans up all datasets in a collection.

### Read downloaded data

With the dataset path from query_collection, we are able to load the data using function read_LUT (meaning read look-up-table) in Indexer. The supported methods are

The first method loads the entire dataset (3D array in the example above); the second method loads the 8th layer of the dataset (only applicable for 3D datasets); the third method returns all the data at a given latitude (30.1°) and longitude (116.1°); and the last method returns only data on the given latitude (30.1°), longitude (116.1°), and cycle index (8). For the third and last methods, option interpolation is false by default and the function returns data that falls into the grid (latitude from 30 to 30.5° and longitude from 116 to 116.5° in this example). If option interpolation is true, we use the bi-linear interpolation method, and the function returns linearly interpolated results from its four nearest neighbours.

### Request partial data

Note that functions query_collection and read_LUT are meant for downloading (if not exist) and reading the local dataset, which may hamper the data reusing. For example, if one only needs the data for a few sites, downloading gigabytes of data for these few sites would (1) be time consuming, (2) waste local storage, and (3) increase unnecessary load for data servers. Therefore, we provide a way to request data for specific sites in Requestor through function request_LUT:

The first method takes the tag name (full name), latitude (30.1°), and longitude (116.1°) as input, and returns the time series of LAI and its error; the second method take an extra cycle index as input, and returns only the data and error for the target cycle (day 57 to 64 in this example). Similar to function read_LUT, one may choose to interpolate the data by setting the interpolation option to false or true. What request_LUT does are (i) user end passes the input information to the server, (2) server end reads the data using query_collection and read_LUT, (3) server end returns a structured JSON file back to the user end, and (4) user end translates the structured JSON back to data (numbers or arrays).

### Other language supports

Besides Julia, we also provide simple user interfaces for Matlab, Octave, Python and R to aid dataset distribution and reuse. We provide three functions for Matlab, Octave, Python and R, and they are (1) update_GM that downloads the latest Artifacts.toml from Github, (2) query_collection that downloads and returns the path of the dataset (same as that in Julia), and (3) request_LUT that requests partial data from the server (same as that in Julia). Different from Julia, update_GM and query_collection download the data to ~/GMCollections rather than ~/.julia/artifacts. Artifacts.toml is stored at ~/GMCollections/Artifacts.toml; the compressed tar.gz files are stored in ~/GMCollections/archives; and the datasets are extracted to ~/GMCollections/artifacts. With the dataset path, users can choose the packages they prefer to read the dataset. In comparison, GriddingMachine.jl updates Artifacts.toml through package releasing, and one needs to update GriddingMachine.jl to use the latest datasets.

### Matlab

We provide Matlab language support via Matlab Toolbox (source code available at https://github.com/Yujie-W/octave-griddingmachine). Matlab users may install and use the toolbox using﻿

### Octave

Octave language support is also provided via https://github.com/Yujie-W/octave-griddingmachine, which is Matlab and Octave compatible. Octave users may install and use the package using﻿

### Python

We provide Python language support via a registered Python package through PyPI (source code available at https://github.com/Yujie-W/python-griddingmachine), and Python users can install the package using pip:

To query the path to downloaded dataset or subset data directly from the server, one may use﻿

### R

We provide R language support via a package hosted on Github, and R users can install and use the package using

As R does not allow for returning multiple variables, data and error of the request are stored as fields of a list, and one can read them out using results$data and results$std.

## Discussion

**Fig. 3:**
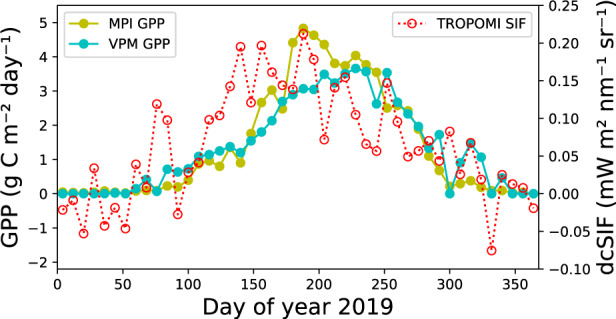
Example of simple dataset requests using GriddingMachine. The requested data are gross primary productivity (GPP) from^27^ (MPI GPP, olive symbols) and^28^ (VPM GPP, cyan symbols) and day length corrected solar-induced chlorophyll fluorescence (dcSIF) from^18^ (TROPOMI SIF, red symbols).

We recommend GriddingMachine users to use provided interfaces through Julia, Matlab, Octave, Python, and R to access the data. In particular, if the research is designed to run at global or large regional scales, we recommend using query_collection to download the datasets, which allows the users to use the latest datasets and perform offline analysis more efficiently. If the research is meant to run at smaller scales such as a few sites, we recommend using request_LUT for convenience. For example, Julia code below shows the simple steps of comparing datasets at the site level. We compared two gross primary productivity products vs. one solar-induced chlorophyll fluorescence for a flux tower site in North America (US-NR1; latitude and longitude are 40.0329° and - 105.5464°, respectively; data from the year 2019), and the result is shown in Fig. 3. In the example presented, requesting the data directly from the server avoids downloading approximately 600 MB reprocessed data (original datasets from three different sources are 2.6 GB):

We note here that the data management of GriddingMachine is subject to future changes and users can follow on the GitHub page at https://github.com/CliMA/GriddingMachine.jl. Users can find tutorials and examples about how to contribute data at https://github.com/CliMA/GriddingMachine.jl#data-contribution.

In conclusion, we present GriddingMachine, a generalized database and software to distribute standardized globally spanning datasets in a user-friendly way. We provide examples that can serve as templates to load different types of datasets we have collected as well an easy way to request partial data from our server in five programming languages: Julia, Matlab, Octave, Python, and R. Given the aims of GriddingMachine, we welcome the contribution of globally gridded data to our collection through https://github.com/CliMA/GriddingMachine.jl/issues/62, and believe that GriddingMachine will be a useful tool for Earth system modeling and ecology communities.

## Methods

To maximally simplify and facilitate the data reuse, we first reprocessed the collected data to standardized NetCDF formatted datasets (Fig. 1). We used NetCDF format because the datasets can be 3D arrays, and the file can be read using non-proprietary software. Then, we compressed each dataset as a tar.gz file, and stored the compressed datasets on public HTTP servers. Last, we stored the meta information in an Artifacts.toml file, and provided user friendly functions to automatically retrieve the datasets for multiple programming languages such as Julia, Matlab, Octave, Python, and R. Note here that Artifacts.toml file is often used in Julia to host meta information of data containers named artifacts. The containers may contain any other kind of data that would be convenient to place within an immutable, life-cycled data store. These containers, (called artifacts) can be created locally, hosted anywhere, and automatically downloaded and unpacked upon request. In GriddingMachine.jl, we used this artifact feature to redistribute the reprocessed datasets, which are not supposed to change with time. Alternatively, users may also download the datasets manually from the HTTP servers.

### Data reprocessing

The raw datasets we collected were in various formats and not standardized (for example, some datasets were scaled but the scaling factors were not noted within the dataset itself). Thus, we reprocessed each dataset before distributing it to the public, and the standards are

- The dataset is stored in a NetCDF file
- The dataset is either a 2D or 3D array
- The dataset is cylindrically projected (WGS84 projection)
- The first dimension of the dataset is longitude
- The second dimension of the dataset is latitude
- The third dimension (if available) is the cycle index, e.g., time
- The longitude is oriented from west to east hemisphere (−180° to 180°)
- The latitude is oriented from south to north hemisphere (- 90° to 90°)
- The dataset covers the entire globe (missing data allowed)
- Missing data is labeled as NaN (not a number) rather than an unrealistic fill value
- The dataset is not scaled (linearly, exponentially, or logarithmically)
- The dataset has common units, such as *μ* mol m^−2^ s^−1^ for maximum carboxylation rate
- The spatial resolution is uniform longitudinally and latitudinally, e.g., both at 1/2°
- The spatial resolution is an integer division of 1°, such as 1/2°, 1/12°, 1/240°
- Each grid cell represents the average value of everything inside the grid cell area (as opposing to a single point in the middle of the cell)
- The label for the data is “data” (for conveniently loading the data)
- The label for the error is “std” (for conveniently loading the error)
- The dataset must contain one data array and one error array besides the dimensions
- The dataset contains citation information in the attributes
- The dataset contains a log summarizing changes if different from original source

Each reprocessed NetCDF file contains four (2D dataset) or five (3D dataset) fields (see Table 2 for the details of the fields and attributes of the reprocessed datasets). We note that there could be infinite number of fields in a NetCDF file; but for the ease of automatically reading the datasets, we only included data and std fields in one reprocessed datasets. For example, the original TROPOMI solar-induced chlorophyll fluorescence (SIF) datasets contain both uncorrected SIF and day length corrected SIF^18,19^, and we partitioned the file to separate files to allow for more automated data requests.

**Table 2:** Fields and attributes of the reprocessed NetCDF datasets.

Field	Dimension	Description	Attributes
lon	1D array	Longitude in the center of a grid	unit°
			description: Latitude
lat	1D array	Latitude in the center of a grid	unit°
			description: Longitude
ind	1D array	Cycle index (only available in 3D datasets)	unit: -
			description: Cycle index
data	2D/3D array	Data in the center of a grid	longname: long name of the data
			unit: unit of the data
			about: general information
			authors: authors of the dataset source publication
			year: year of the data source publication
			title: title of of the data source publication
			journal: journal of the data source publication
			doi: DOI tag of the data source publication
			changeN: Change log of the *N* th change we made
std	2D/3D array	Error of data in the center of a grid	same as “data” field

### Data packaging

After data reprocessing, we compressed each dataset along with an empty GRIDDINGMACHINE file as a tar.gz file (contains 2 files). The empty GRIDDINGMACHINE file labels the NetCDF file as a GriddingMachine dataset (used to clean up outdated datasets). For example, any folder with the empty file GRIDDINGMACHINE will be treated as a GriddingMachine artifact, and the hashtag for this artifact is the same as the folder name. If this hashtag does not exist in Artifacts.toml, then this GriddingMachine artifact will be marked as outdated. Users can delete the outdated GriddingMachine using clean_collections!. The NetCDF dataset file name and the tag name are the same and follow a general naming pattern: LABEL_(EXTRALABEL_)IX_JT_(YEAR_)VK. See Table 1 for current collections and https://github.com/CliMA/GriddingMachine.jl/issues/62 for the growing collection list and detailed change logs for each dataset.

Meta information of all the datasets is stored in Artifacts.toml, and each item of the toml file includes the following: tag name, SHA1 hash value, SHA256 hash value, and downloading URLs. Here SHA stands for Secure Hash Algorithm, and it was used to compute a unique hashtag for each dataset, thus aiding data verification when users use the data. Through Artifacts.toml, we provided functions to automatically download and load the datasets in multiple programming languages.

## Data Availability

The compressed datasets can be downloaded from CaltechDATA^17^.

## Code availability

The code can be found at https://github.com/CliMA/GriddingMachine.jl under the Apache 2.0 License. The exact version of the package used to produce the results presented in this paper is also archived on CaltechDATA along with the datasets^17^.
